# Correction to “Programmed Death Ligand 1 Regulates Epithelial–Mesenchymal Transition and Cancer Stem Cell Phenotypes in Hepatocellular Carcinoma Through the Serum and Glucocorticoid Kinase 2/β‐Catenin Signaling Pathway”

**DOI:** 10.1111/cas.70316

**Published:** 2026-01-05

Kong X, Peng H, Liu P, Fu X, Wang N, Zhang D. Programmed death ligand 1 regulates epithelial–mesenchymal transition and cancer stem cell phenotypes in hepatocellular carcinoma through the serum and glucocorticoid kinase 2/β‐catenin signaling pathway. *Cancer Sci*. 2023; 114: 2265–2276. https://doi.org/10.1111/cas.15753

In the above article, the MHCC97H siRNA‐con image in Figure 4D was incorrect. The correct image for Figure 4 is shown below

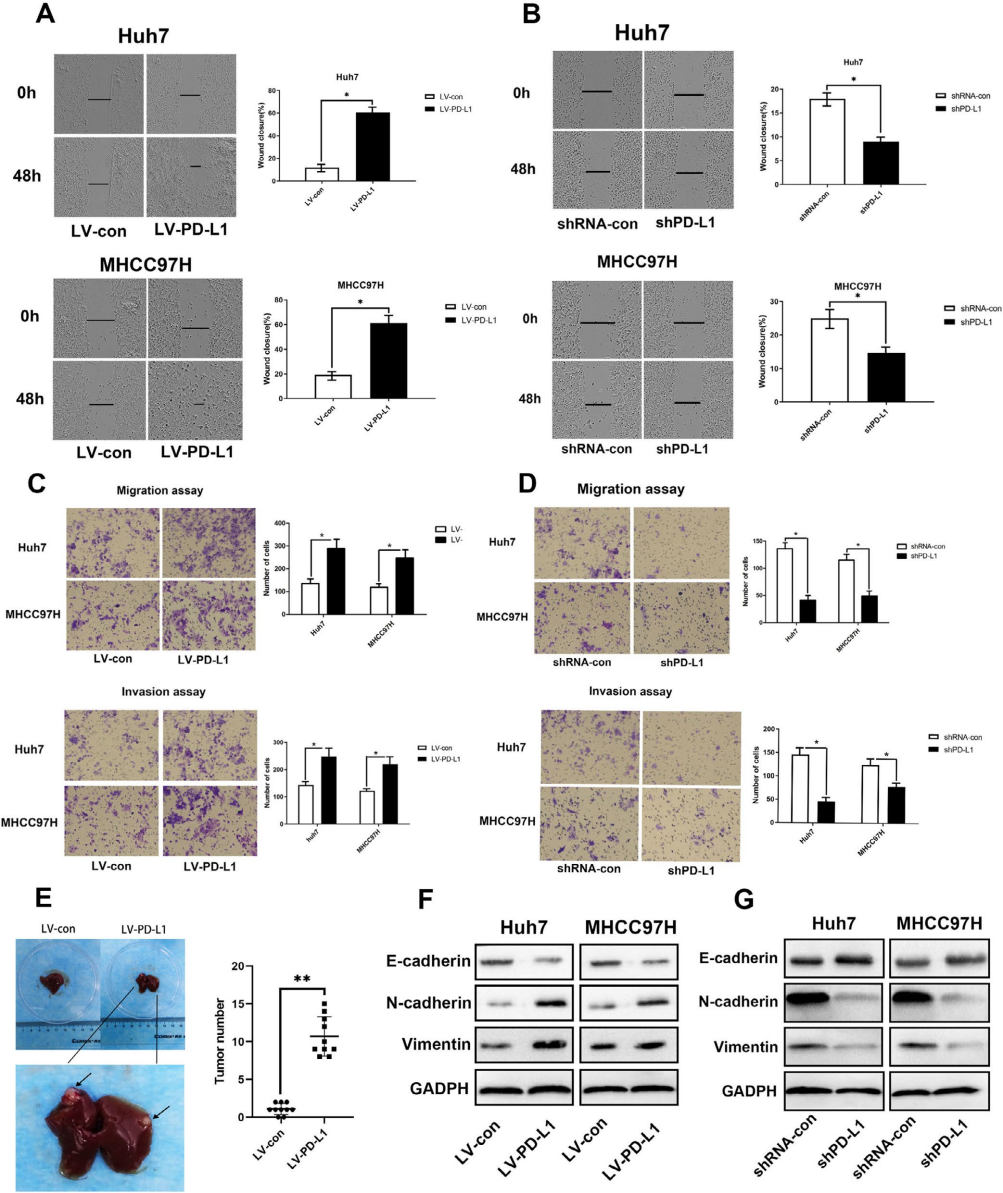

We apologize for this error.

